# miR-21 in the Extracellular Vesicles (EVs) of Cerebrospinal Fluid (CSF): A Platform for Glioblastoma Biomarker Development

**DOI:** 10.1371/journal.pone.0078115

**Published:** 2013-10-21

**Authors:** Johnny C. Akers, Valya Ramakrishnan, Ryan Kim, Johan Skog, Ichiro Nakano, Sandeep Pingle, Juliya Kalinina, Wei Hua, Santosh Kesari, Ying Mao, Xandra O. Breakefield, Fred H. Hochberg, Erwin G. Van Meir, Bob S. Carter, Clark C. Chen

**Affiliations:** 1 Center for Theoretical and Applied Neuro-Oncology, University of California, San Diego, California, United States of America; 2 Exosome Diagnostics, New York, New York, United States of America; 3 Dardinger Laboratory for Neurosciences, Department of Neurosurgery, Ohio State University,Columbus, Ohio, United States of America; 4 Department of Neurosciences, University of California, San Diego, California, United States of America; 5 Neurology Service, Massachusetts General Hospital, and Program in Neuroscience, Harvard Medical School, Boston, Massachusetts, United States of America; 6 Department of Neurosurgery and Hematology & Medical Oncology, School of Medicine and Winship Cancer Institute, Emory University, Atlanta, Georgia, United States of America; 7 Department of Neurosurgery, Huashan Hospital, Fudan University, Shanghai, China; City of Hope, United States of America

## Abstract

Glioblastoma cells secrete extra-cellular vesicles (EVs) containing microRNAs (miRNAs). Analysis of these EV miRNAs in the bio-fluids of afflicted patients represents a potential platform for biomarker development. However, the analytic algorithm for quantitative assessment of EV miRNA remains under-developed. Here, we demonstrate that the reference transcripts commonly used for quantitative PCR (including GAPDH, 18S rRNA, and hsa-miR-103) were unreliable for assessing EV miRNA. In this context, we quantitated EV miRNA in absolute terms and normalized this value to the input EV number. Using this method, we examined the abundance of miR-21, a highly over-expressed miRNA in glioblastomas, in EVs. In a panel of glioblastoma cell lines, the cellular levels of miR-21 correlated with EV miR-21 levels (p<0.05), suggesting that glioblastoma cells actively secrete EVs containing miR-21. Consistent with this hypothesis, the CSF EV miR-21 levels of glioblastoma patients (n=13) were, on average, ten-fold higher than levels in EVs isolated from the CSF of non-oncologic patients (n=13, p<0.001). Notably, none of the glioblastoma CSF harbored EV miR-21 level below 0.25 copies per EV in this cohort. Using this cut-off value, we were able to prospectively distinguish CSF derived from glioblastoma and non-oncologic patients in an independent cohort of twenty-nine patients (Sensitivity=87%; Specificity=93%; AUC=0.91, p<0.01). Our results suggest that CSF EV miRNA analysis of miR-21 may serve as a platform for glioblastoma biomarker development.

## Introduction

Glioblastoma is the most common form of primary brain cancer and remains one of the deadliest of human cancers [1]. Timely diagnosis and sensitive therapeutic monitoring remain major challenges in the treatment of this disease. Clinically, response evaluations are largely based on clinical examination and Magnetic Resonance Imaging (MRI) [2]. However, both clinical examination and MRI are insensitive measures of disease status. For instance, the lowest resolution for reliable detection by MRI is on the order of millimeters [3]. Considering the size of a tumor cell, this limitation in resolution translates into a delay of at least ten cell divisions before therapeutic resistance can be detected [4]. While repeated post-treatment biopsies constitute a monitoring option, this practice is associated with significant morbidity [5,6]. In this context, less invasive platforms for therapeutic monitoring are needed. 

Recent studies suggest that glioblastoma cells secrete extracellular vesicles (EVs) containing genetic materials that mirror the intracellular tumor milieu, including tumor specific mutations and patterns of RNA expression [7–11]. These EVs are released into the local environment and transgress anatomic compartments into cerebrospinal fluid (CSF) and the systemic blood circulation [7,8,12,13]. Analyses of these EVs offer a potential platform for monitoring tumor presence, phenotypic/genotypic features, and pathophysiology [7,9,14,15]. The EVs shelter the tumor-specific genetic material from the extracellular environment that is replete with RNAses [16,17] and preserve the integrity of these materials. Importantly, the genetic materials within EVs appear highly enriched for RNAs in the size range of microRNAs (miRNAs) [9].

Since non-neoplastic cells secrete EVs [18–20] and they out-number neoplastic cells by several orders of magnitude in a patient [21], tumor-specific EVs remain a rarity in clinical samples [22]. As such, sensitive methods of amplification, such as quantitative polymerase chain reactions (qPCR), are required for detection and quantitation of RNA in EVs [7,8,23]. In the frequently used relative C_T_ method of qPCR [24], the cellular abundance of a “query” transcript is assessed by determining the number of cycles of PCR required to reach a threshold quantity [25]. To correct for the quality and quantity of the input material, this cycle number is then normalized to the PCR cycles required for a reference transcript to reach the same threshold [26]. This method assumes abundance and near-homogeneity of the reference transcript level per cell [27,28]. 

Here we report that the commonly used reference transcripts for quantitation of cellular RNA, including GAPDH, 18S rRNA, and hsa-miR-103, are present in extremely low and highly varied levels within EVs (on the order of 1 copy per 10^3^ to 10^4^ EVs). Moreover, their relative abundance in EVs bears no correlation to the total EV number or RNA yield. In this context, we characterized the abundance of EV miRNAs in absolute terms and normalized this value to the input EV particle numbers. Using this method, we studied the level of hsa-miR-21, a miRNA that is highly over-expressed in glioblastoma cells [29]. Our results indicate that miR-21 levels were significantly elevated in EVs isolated from the CSF of glioblastoma patients relative to those derived from non-oncologic patients.

## Materials and Methods

### Clinical specimen collection

All research performed were approved by IRB boards at University of California, San Diego Human Research Protections Program and were in accordance with the principles expressed at the declaration at Helsinki. Each patient was consented by a dedicated clinical research specialist prior to collection. Written consent was obtained for each patient. The consent process was approved by the ethics committee, and all records were documented in our electronic record system. The written consent from patients was also scanned into our electronic filing system. The serum and CSF specimens for the initial, exploratory studies were collected at the University of California San Diego Medical Center under IRB 120345X. The sera collection and an initial training CSF collection were performed by CCC and BSC at the time of surgical procedure. The CSF was collected by ventricular/lumbar drain placement or cisternal aspiration at the time of craniotomy. Blood was collected using an 18 Gauge-needle venipuncture into clot-activating blood collection tubes with gel separator (BD vacutainer catalog #366450). Attention was paid to minimize mechanical tube agitation. The samples were processed by spinning at 1,500 x g within 30 minutes of collection and the snap frozen [30]. The clinical diagnosis of the non-oncologic patients who contributed blood samples were: severe head trauma (n=2), subarachnoid hemorrhage (n=2), and normal pressure hydrocephalus (n=1). The clinical diagnosis of the non-oncologic patients who contributed CSF samples were: trauma (n=2), subarachnoid hemorrhage (n=8), normal pressure hydrocephalus (n=2), arteriovenous malformation (n=2). All diagnoses of glioblastoma were histologically confirmed. CSF specimens were snap frozen in -80 upon receipt and analyzed without further centrifugation. The CSF specimens for the validation study were generously provided by Dr. Santosh Kesari (University of California, San Diego; IRB 110551X) and Dr. Erwin Van Meri (Emory University, IRB 642-2005). 1-5 mL of CSF was collected from each patient through lumbar or ventricular puncture.

### EV free media preparation

EV-depleted medium was prepared by ultracentrifugation of DMEM supplemented with 20% FBS at 120,000 x g for 18 hours at 4°C. The medium was then diluted to a final concentration of 10% FBS and used to culture cell lines as described.

### Cell lines and cell culture

Eleven human glioblastoma cell lines (A1207, A172, LN18, LN340, LN464, T98G, U118, U373, U87MG, LN229, and LN235) [31] and 3 non-glioblastoma cell lines (A549, U20S, and 293T) [32–34] were cultured in DMEM supplemented with 10% FBS. At 60-70% confluency, the standard culture medium was replaced with EV depleted medium. The cells were cultured for an additional 72 hours before EV collection from the cell-free supernatants. 9 neurosphere lines (1123, 326, 83, 30, AC17, AC20, 84, BT70, and CMK3) were cultured in DMEM F12 supplemented with growth factors as described previously [35–37]. Cell free supernatant was collected three days after culturing for EV isolation.

### Extracellular vesicle (EV) Isolation

The effect of freeze-thaw cycle on the stability of vesicular content has not been well studied. In order to avoid degradation of vesicles, all samples were processed promptly upon thawing of serum and CSF samples. The EV fraction was isolated by differential centrifugation [38]. Briefly, conditioned media or diluted bio-fluids were first centrifuged at 2,000 x g for 20 minutes to remove cellular debris. The supernatant was collected and further centrifuged at 10,000 x g for 30 minutes. The resultant supernatant was then transferred to ultracentrifuge tubes for ultracentrifugation at 120,000 x g for 2 hours. The supernatant was discarded and the EV pellets were re-suspended in 150μL of PBS for storage at -80°C prior to RNA isolation. All centrifugation steps were performed at 4°C. This protocol was designed to enrich for EVs in the 50-250 nm size range (Figure **5**). 

### EV Quantification and Assessment

**Figure 1:**
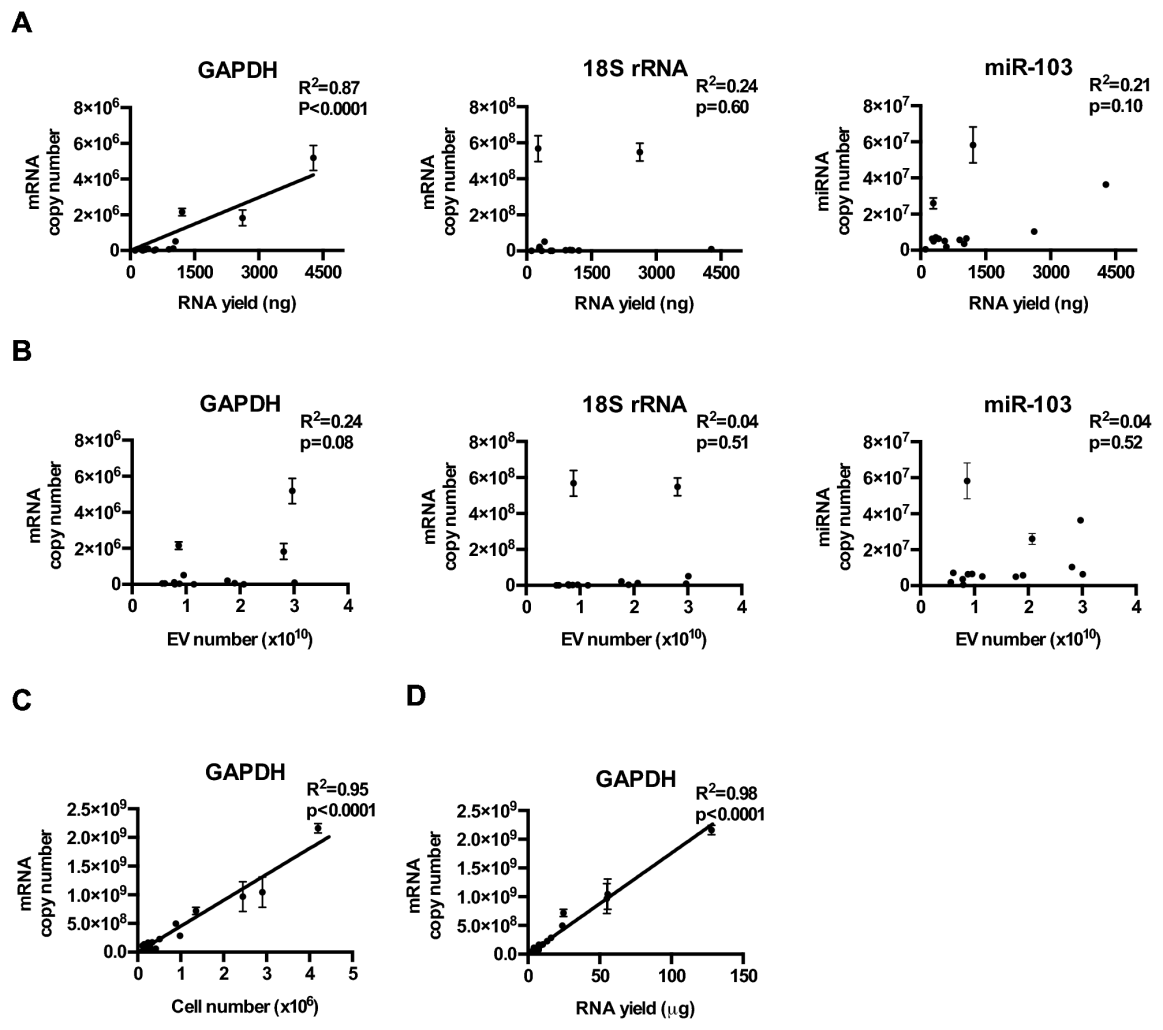
Relative abundance of GAPDH, 18S rRNA, and miR-103 in glioblastoma cell line derived EVs. (**A**) GAPDH, 18S rRNA, and hsa-mir-103 levels in EVs isolated from 11 glioblastoma and 3 non-glioblastoma adherent cell lines, cultured under serum conditions. Transcript copy number was plotted against total RNA yield extracted from the EVs. (**B**) GAPDH, 18S rRNA, and hsa-mir-103 transcript copy number was plotted against the total number of EVs isolated for RNA extraction. (**C**) Cellular GAPDH transcript number tightly correlated with the number of cells collected for RNA extraction and (**D**) the amount of RNA recovered.

The number of vesicles recovered was determined by Nanoparticle Tracking Analysis (NTA) on a Nanosight LM-10HS in accordance to the manufacturer’s instructions (Nanosight, Wiltshire, UK). Resuspended vesicles were diluted 1:40 to 1:200 with PBS before analysis. The purity of the EV isolated were assessed using electron microscopy as previously described [38] (Figure **1**).

### Quantitative Reverse Transcriptase-Polymerase Chain Reaction (qRT-PCR)

RNA from the EV fraction was extracted using the mirRCURY RNA Isolation Kit (Exiqon, Vedbaek, Denmark) per manufacturer’s protocol. RNA concentration and quality were determined using the NanoDrop ND-1000 Spectrophotometer (Thermo Scientific, Waltham, MA). For profiling GAPDH, 18S rRNA, miR-21, and miR-103 expression, cDNA was synthesized with the miRCURY LNA^TM^ Universal RT microRNA PCR system (Exiqon). The resultant cDNA were diluted 20x for qRT-PCR. (See Table **1** for primer information). 

### Determination of copy number

To determine the absolute copy numbers of GAPDH and 18S RNA in the cellular cytoplasm and in the EVs, a standard curve for each gene was generated using serial dilutions of known quantity of U87MG genomic DNA. The copy number of GAPDH and 18S RNA within the U87 genome was previously determined by integrated sequencing efforts and comparative genomic hybridization [39]. To determine copy number of miRNA (miR-103, miR-21), standard curves were generated by serial dilution of known quantities of miRNA mimic (Qiagen, Germantown, MD), followed by cDNA synthesis using the miRCURY LNA^TM^ Universal RT microRNA PCR system under conditions that allow one round of cDNA synthesis [40]. The cDNAs were then used for the generation of standard curve.

### Statistical Analysis

All statistical analyses were performed using the GraphPad Prism Software version 5 (GraphPad, La Jolla, CA). Correlation between GAPDH, 18S RNA, and miR-103 transcript level, EV RNA yield, and EV numbers were determined using Pearson’s Correlation [41]. Difference in hsa-miR-21 level between glioblastoma and non-oncologic clinical samples was determined using the student’s t-test (2 tailed) [42]. Sensitivity, specificity, positive predictive value (PPV), and negative predictive value (NPV), Receiver Operating Characteristic (ROC) curve and Area Under the Curve (AUC) were determined as previously described [43]. 

## Results

### GAPDH, 18S rRNA, and hsa-miR-103 levels in glioblastoma cell line derived EVs

Housekeeping genes, such as GAPDH, 18S rRNA, and miR-103, are expressed at high levels across cell types and exhibit little cell-to-cell variability relative to most query genes. As such, they are considered well-validated reference genes in the expression profiling of cellular contents [27,28,44]. The absolute copy number of these transcripts in EVs, however, remains poorly characterized. Since the mechanisms by which genetic materials are transported from the cell into the EVs remain largely unknown [45], it is conceivable that EV transcript levels differ from their cellular expression levels. Despite this knowledge gap, GADPH mRNA, 18s rRNA, and miR-103 transcripts are frequently used as reference genes for quantitative analysis of EV-derived genetic materials [46–48] via the relative C_T_ qPCR method [49–53]. 

We first determined the relative abundance of GAPDH mRNA, 18S rRNA, and hsa-miR-103 in EVs derived from 6 glioblastoma and 2 non-glioblastoma adherent cell lines. All three transcripts were abundant in the cellular cytoplasm, with copy numbers that ranged from 300 to 1,000 copies per cell for GAPDH, 100,000 to 1,000,000 copies for 18S rRNA, and 8,000 to 80,000 copies for hsa-miR-103 (Table **1**). These copy numbers are comparable to those previously published [54–57]. 

**Table 1:** Abundance of GADPH mRNA, 18s RNA, and hsa-miR-103 in cultured cell lines and EVs derived from cultured cell lines.

		**GAPDH**		**18S RNA**		**miR-103**	
	**Cell line**	**Copies/cell**	**Copies per EV**	**Copies/cell**	**Copies per EV**	**Copies/cell**	**Copies per EV**
Adherent lines	U87MG	6.97 x 10^2^	1.14 per 10^5^	1.29 x 10^6^	1.71 per 10^3^	8.31 x 10^3^	2.13 per 10^4^
	T98G	3.21 x 10^2^	2.02 per 10^6^	2.80 x 10^5^	1.87 per 10	4.42 x 10^4^	7.23 per 10^4^
	LN229	4.52 x 10^2^	1.46 per 10^3^	1.09 x 10^5^	1.81 per 10^2^	1.02 x 10^5^	6.74 per 10^3^
	LN464	3.27 x 10^2^	8.63 per 10^7^	1.21 x 10^6^	6.36 per 10^4^	8.65 x 10^4^	1.26 per 10^3^
	A172	2.91 x 10^2^	2.58 per 10^6^	9.32 x 10^5^	1.35 per 10^4^	1.90 x 10^4^	4.47 per 10^4^
	LN340	5.10 x 10^2^	6.24 per 10^5^	5.13 x 10^5^	1.28 per 10^3^	2.58 x 10^4^	2.82 per 10^4^
	A549	1.03 x 10^3^	1.30 per 10^4^	1.34 x 10^6^	3.19 per 10^4^	3.92 x 10^4^	3.35 per 10^4^
	U2OS	9.06 x 10^2^	1.84 per 10^4^	1.41 x 10^6^	3.84 per 10^4^	7.42 x 10^4^	6.79 per 10^4^
Neurospheres	1123	3.96 x 10^2^	3.93 per 10^6^	3.99 x 10^6^	5.75 per 10^5^	3.68 x 10^3^	2.47 per 10^4^
	326	5.14 x 10^2^	3.36 per 10^5^	6.36 x 10^6^	6.38 per 10^3^	2.13 x 10^3^	6.95 per 10^4^
	83	5.62 x 10^2^	6.39 per 10^6^	6.63 x 10^6^	2.63 per 10^3^	2.15 x 10^3^	9.23 per 10^5^
	30	3.60 x 10^2^	9.24 per 10^6^	4.66 x 10^6^	1.74 per 10^3^	5.16 x 10^3^	8.42 per 10^4^
	AC17	2.92 x 10^2^	9.00 per 10^6^	9.96 x 10^5^	3.85 per 10^5^	3.24 x 10^3^	6.82 per 10^5^
	AC20	1.42 x 10^2^	1.08 per 10^5^	6.13 x 10^5^	5.01 per 10^4^	7.62 x 10^3^	4.03 per 10^4^
	84	2.37 x 10^2^	2.93 per 10^5^	1.03 x 10^6^	1.40 per 10^3^	3.41 x 10^3^	8.16 per 10^5^
	BT70	5.30 x 10^2^	5.43 per 10^5^	2.23 x 10^6^	3.88 per 10^4^	4.11 x 10^3^	3.36 per 10^5^
	CMK3	2.12 x 10^2^	2.95 per 10^4^	6.72 x 10^5^	2.41 per 10^3^	6.53 x 10^3^	1.75 per 10^4^

We next analyzed the level of reference transcripts in EVs secreted by these adherent cell lines. EVs were isolated by ultracentrifugation and their numbers were determined by Nanoparticle Tracking Analysis [58–61]. While all three transcripts were detectable, their abundance in the EVs were approximately 10^5^ -10^10^ fold lower than the levels detected in the cellular cytoplasm. At most, an average of one copy of GAPDH transcript was detected in 1,000 EVs; an average of one 18S rRNA transcript was found in 40 EVs; an average of one copy of miR-103 transcript was present in 750 EVs (Table **1**). 

To determine whether reference housekeeping genes would serve as surrogate markers for the total RNA yield or the total number of EVs, we analyzed the levels of GAPDH mRNA, 18S rRNA, and hsa-miR-103 in EVs isolated from 11 glioblastoma and 3 non-glioblastoma adherent cell lines (cultured under serum conditions). We observed that the level of the potential reference transcripts varied by at least an order of magnitude in EVs isolated from different lines. The expression of the 18S rRNA and hsa-miR-103 demonstrated low correlation with EV RNA yield (Figure **1A**) or EV number (Figure **1B**). While EV GADPH transcript level demonstrated correlation with EV RNA yield and number, these correlations were not as robust as those seen between cellular GAPDH transcript level and cell number (Figure **1C**) or the RNA yield from cell extracts (Figure **1D**). 

**Figure 2:**
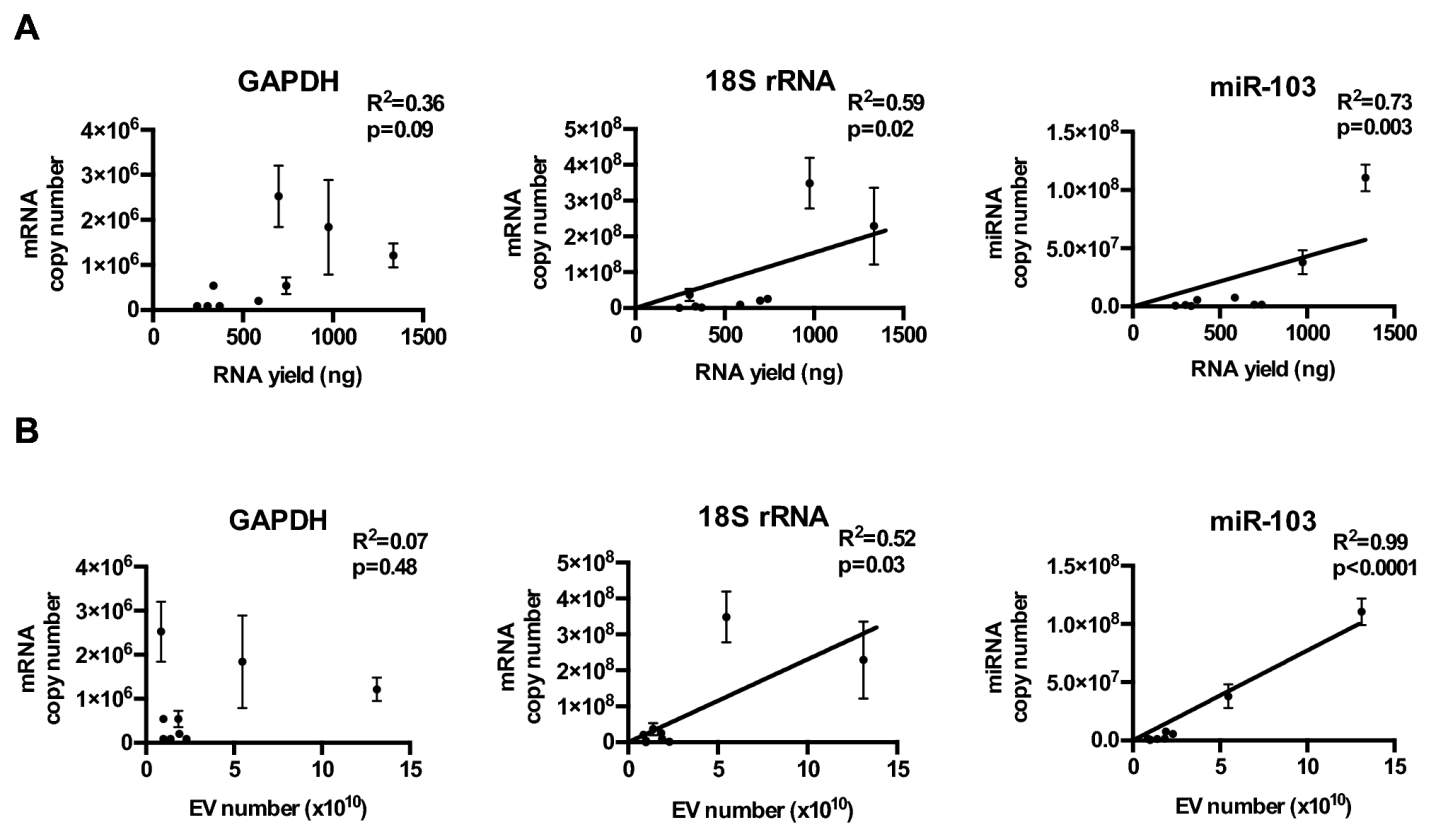
Relative abundance of GAPDH, 18S rRNA, and miR-103 in neurosphere glioblastoma line derived EVs. (**A**) GAPDH, 18S rRNA, and hsa-mir-103 levels in EVs isolated from 9 glioblastoma neurophere lines, cultured under serum-free conditions. Transcript copy number was plotted against total RNA extracted from the EVs. (**B**) GAPDH, 18S rRNA, and hsa-mir-103 transcript copy number was plotted against the total number of EVs isolated for RNA extraction.

**Figure 3:**
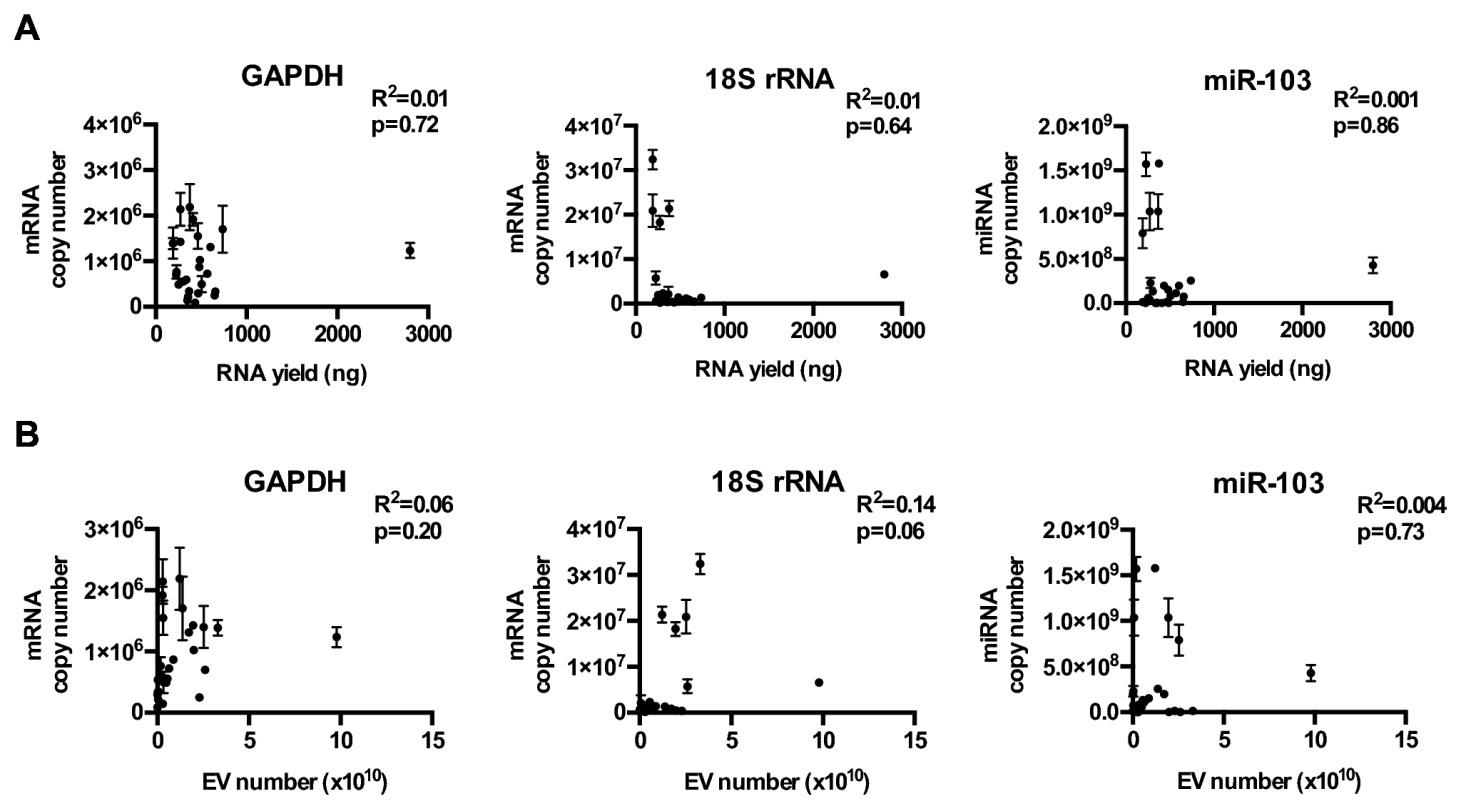
Relative abundance of GAPDH, 18S rRNA, and miR-103 in EV derived from CSF of glioblastoma and non-oncologic patients. (**A**) GAPDH, 18S rRNA, and hsa-mir-103 levels in EVs isolated from the CSF of 13 glioblastoma and 14 non-oncologic patients. Transcript copy number was plotted against total RNA extracted from the EVs. (**B**) GAPDH, 18S rRNA, and hsa-mir-103 transcript copy number was plotted against the total number of EVs isolated for RNA extraction. The relative abundance of these transcripts in CSF EVs was approximately 10 fold lower than those of sera EV with abundance ranging between 1 transcript in 1 EV to 1 transcript in 105 EVs.

As cell lines cultured under neurosphere and serum-free conditions may better recapitulate certain aspects of glioblastoma biology [62,63], we quantitated the three potential reference transcripts in the EVs isolated from nine glioblastoma neurosphere lines [35–37]. While GAPDH mRNA, 18S rRNA, and hsa-miR-103 were abundant in the cellular cytoplasm, their abundance in the EVs was 10^8^ fold less relative to the cellular content (Table **1**). In contrast to the results obtained using adherent glioblastoma lines, GADPH mRNA levels in EVs derived from neurosphere lines did not correlate with EV RNA yield (Figure **2A**) or EV number (Figure **2B**). Instead, correlations were observed between EV hsa-miR-103 and 18s rRNA and EV number/EV RNA yield (Figures **2A and 2B**). Careful analysis of the correlation plot, however, suggests that the correlations may have been driven by a small subset of samples. In EVs derived from cultured cells, there was a strong agreement between RNA yield from EV preparation and the total EV number (R^2^=0.74, p=0.003) (Figure **3A**).

### GADPH mRNA, 18S rRNA, and hsa-miR-103 levels in EVs derived from patient sera and cerebrospinal fluid (CSF)

We next tested whether the abundance of GAPDH mRNA, 18S rRNA, and hsa-miR-103 correlated with RNA yield or EV number in clinical specimens from glioblastoma patients. The purity of the EVs isolated from clinical sera or CSF was verified by electron microscopy (Figure **1**). There was no convincing or consistent correlation between GADPH, 18s rRNA, or miR-103 transcripts and EV particle number in serum (Figure **2**) or CSF (Figure **3**). There was little correlation between EV number and RNA yield in EVs derived from sera derived EVs (R^2^=0.14, p=0.04). Modest correlation between RNA yield and EV number was observed in CSF derived EVs (R^2^=0.60, p<0.001; Figure **3B and C**)**.**

In summary (Table **2**), EV GAPDH mRNA levels correlated with EV numbers in glioblastoma lines cultured under adherent conditions, but this correlation was not observed in lines cultured under neurosphere conditions. On the other hand, EV miR-103 and 18s RNA levels correlated with EV number in glioblastoma lines cultured under neurosphere conditions, but not in lines cultured under adherent conditions. In clinical sera or CSF, there was no correlation between these reference transcripts and EV numbers. These discrepancies create significant uncertainties as to the appropriate reference transcript for quantitative EV analysis in clinical bio-fluids. Given these discrepancies and the lack of correlation between EV GAPDH mRNA, 18S rRNA, or miR-103 and EV number/EV RNA yield, we propose that EV RNA be quantitated in absolute terms and normalized to the input EV number. We reasoned that this method is analogous to determining absolute quantities of cellular miRNA and normalizing to the input cell number. Our proposed method prevents arbitrary changes in miRNA quantitation secondary to random fluctuation in the levels of the reference transcript. 

**Table 2:** Overview of robustness of reference transcripts in EVs derived from cell lines and bio-fluids.

	**Correlation with RNA yield**			**Correlation with EV number**		
**Sample type**	**GAPDH**	**18S rRNA**	**miR-103**	**GAPDH**	**18S rRNA**	**miR-103**
Adherent lines	**+**	**-**	**-**	**-**	**-**	**-**
Neurosphere lines	**-**	**+**	**+**	**-**	**+**	**+**
Serum	**NT**	**NT**	**NT**	**-**	**-**	**-**
Cerebrospinal Fluid	**-**	**-**	**-**	**-**	**-**	**-**

No correlation

+ Significant correlation (R^2^ >0.5 and p<0.05)

NT: Not testedmiR-21 levels in EVs derived from glioblastoma cell lines

Using absolute measurements of miRNA normalized to input EV numbers, we characterized the levels of miR-21, a highly over-expressed miRNA in glioblastoma [29], in EVs isolated from glioblastoma neurosphere lines. We reasoned that if EV miRNA contents reflect the internal milieu of the secreting cell [34], then miR-21 should be one of the more abundant species in glioblastoma EVs. Indeed, microarray based miRNA profiling of EVs derived from two glioblastoma cell lines revealed that miR-21 is one of the most abundant miRNAs in glioblstoma EVs [7].

**Figure 4:**
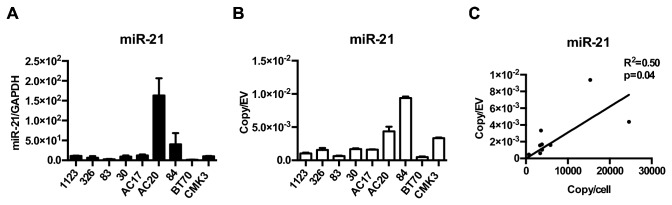
Detection of miR-21 in glioblastoma cell lines secreted EVs. (**A**) The expression level of miR-21 was quantitatively assessed in nine neurosphere glioblastoma lines. (**B**) The abundance of miR-21 was assessed in EVs derived from nine neurosphere glioblastoma lines. (**C**) EV miR-21 levels correlate tightly with cellular miR-21 levels (R^2^ = 0.50, p<0.01).

We first characterized EV miR-21 using nine glioblastoma neurosphere lines. Consistent with published literature, miR-21 was highly expressed in these lines, with quantities that ranged from 700 to 20,000 copies per cell (Figure **4A**, Table **3**). miR-21 levels per EV were significantly lower, ranging from 1 copy per 100 to 2,000 EVs (Figure **4B**). Nevertheless, miR-21 was consistently detectable in EVs isolated from all nine lines. Moreover, the EV miR-21 level correlated with the cellular miR-21 level (R^2^=0.50; p<0.05), suggesting that EV miRNA analysis may afford a window into the physiology of the secreting cell (Figure **4C**). 

**Table 3:** Abundance of miR-21 in cultured neurosphere lines and neurosphere derived EVs.

**miR-21**		
**Cell line**	**Copies/cell**	**Copies per vesicle**
1123	3.87 x 10^3^	1.05 per 10^3^
326	3.36 x 10^3^	1.57 per 10^3^
83	3.40 x 10^3^	6.28 per 10^4^
30	3.83 x 10^3^	1.68 per 10^3^
AC17	5.86 x 10^3^	1.60 per 10^3^
AC20	2.46 x 10^4^	4.37 per 10^3^
84	1.54 x 10^4^	9.38 per 10^3^
BT70	7.14 x 10^2^	4.80 per 10^4^
CMK3	3.60 x 10^3^	3.34 per 10^3^

 We also examined the effect of the culturing condition on the abundance of miR-21 in EVs. To this end, the CMK-3 neurosphere cell line was adapted to growth as adherent culture in the presence of fetal bovine serum. This experimental manipulation led to a nine-fold increase in the cellular level of miR-21. However, the abundance of miR-21 in secreted EVs was decreased by approximately ten fold (Figure **6**). In a reciprocal experiment, adherent U87MG cells were cultured under neurosphere conditions. This adaptation led to decreased abundance of miR-21 in both cellular and EV contents. These results suggest that glioblastoma growth under neurosphere conditions is associated with decreased cellular levels of miR-21. While culturing conditions also significantly impact EV miR-21 level, these effects are less predictable.

### miR-21 levels in EVs isolated from glioblastoma and non-oncologic patients

Since miR-21 is present in glioblastoma secreted EVs, if these EVs eventually reach bio-fluids such as blood or CSF, then the elevated levels of EV miR-21 in these bio-fluids may serve to indicate the presence of tumor. This hypothesis pre-supposes that EVs secreted from normal cells harbor significantly lowered levels of miR-21 relative to those of glioblastoma EVs. As a first step toward testing this hypothesis, we determined whether miR-21 levels in clinical bio-fluids (sera and CSF) derived from glioblastoma patients differed from those of non-oncologic patients. 

**Figure 5:**
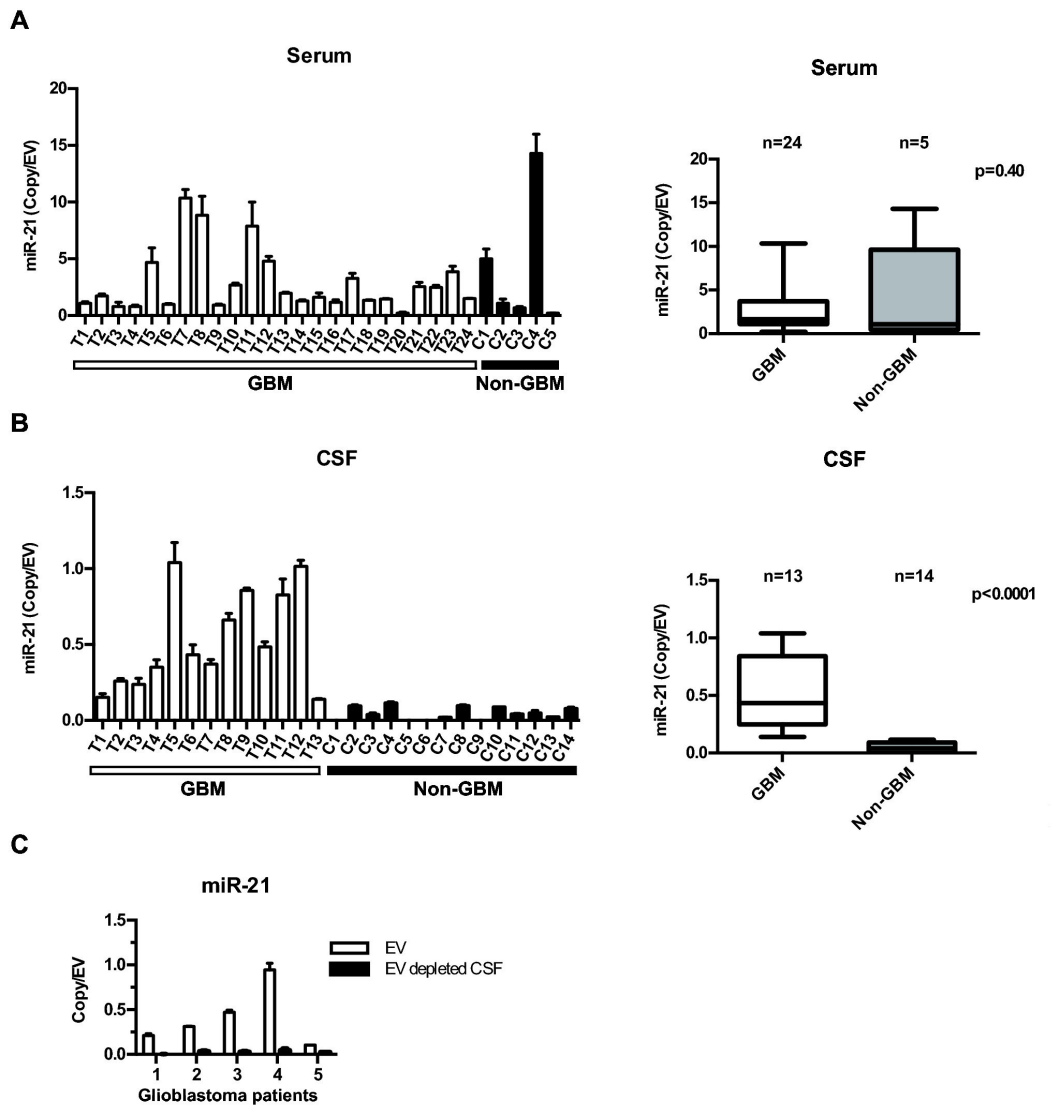
Discrimination of glioblastoma disease status by CSF EV miR-21 analysis. (**A**) Comparable levels of miR-21 level in sera EVs derived from 24 glioblastoma patients and 5 non-oncologic patients. (**B**) Elevated level of miR-21 in CSF EV derived from 13 glioblastoma patients relative to 14 non-oncologic patients. (**C**) miR-21 is detected in CSF EV but not in EV depleted CSF in samples isolated from five independent glioblastoma patients. Patient 1, 2, 3, 4, and 5 correspond to T2, T7, T10, T12, and T13 respectively from the UCSD glioblastoma cohort.

Since EVs bearing tumor specific RNAs have been detected in blood [7], we tested whether miR-21 levels differed in EVs derived from the sera of 24 glioblastoma patients differed from those of 5 non-oncologic patients (2 trauma, 2 subarachnoid hemorrhage, 1 normal pressure hydrocephalus). On average, 1.96x10^9^ to 1.19x10^11^ EVs were isolated from 2-5cc of sera. The level of EV miR-21 ranged from 0.21 to 10.35 copies/EV in the glioblastoma patients and 0.21 to 14.30 copies/EV in the non-oncologic patients. No statistically significant difference was detected between these two groups (Figure **5A**).

We hypothesized that the secretion of EV miR-21 by hematopoietic cells [64] might obscure glioblastoma EV miR-21 in sera. Thus, we analyzed miR-21 levels in EVs from CSF of glioblastoma (n=13) and non-oncologic patients (n=14). CSF EV miR-21 ranged from 0.14 to 1.04 copies/EV in glioblastoma patients and 5.26x10^-4^ to 1.48x10^-1^ copies/EV in non-oncologic patients. The difference between these two groups was statistically significant at p<0.001 (Figure **5B**), suggesting that CSF EV may be a platform for therapeutic monitoring of tumor presence. We also noted that the bulk of miR-21 is located within EVs rather than freely floating in CSF, <10% of total CSF miR-21 (Figure **5C**) was detected in the EV depleted CSF fraction in samples collected from five independent glioblastoma patients. In our analysis, we noted that all of the CSFs derived from non-oncologic patients harbored EV miR-21 level of < 0.25 copy/EV. Of note, the number of EVs as well as the size distribution of EVs isolated from the bio-fluids of glioblastoma patients did not significantly differ from those isolated from non-oncologic patients (Figures **4** and **5**).

### CSF EV miR-21 analysis in an independent cohort

**Figure 6:**
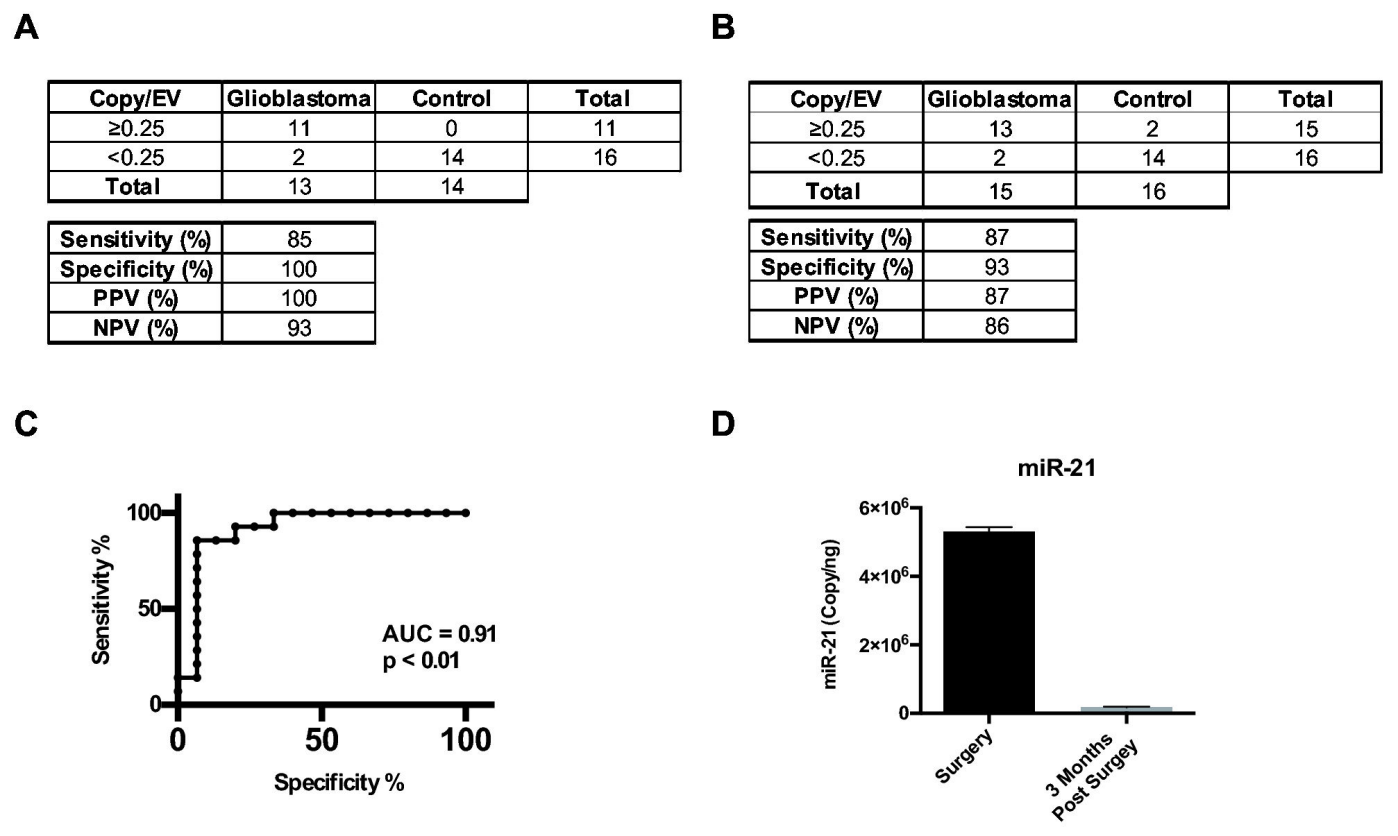
Elevated levels of CSF EV miR-21 are detected in glioblastoma patients. (**A**) Sensitivity, specificity, positive predictive, and negative predictive values associated with EV miR-21 level of <0.25 copy/EV as a discriminating threshold for glioblastoma disease status in the initial exploratory study. (**B**) Sensitivity, specificity, positive predictive, and negative predictive values associated with EV miR-21 level of <0.25 copy/EV as a discriminating threshold for glioblastoma disease status in a validation study. (**C**) Receiver Operating Characteristic Curve for EV miR-21 level of <0.25 copy/EV as a discriminating threshold for glioblastoma disease status. (D) Level of miR-21 in CSF EV from a glioblastoma patient at time of surgery and three months after a gross-total resection. The relative abundance of miR-21 in CSF EV was decreased by approximately 50-fold after surgical resection.

We next wished determined whether such a threshold can be utilized to discriminate CSF derived from glioblastoma patients from those of non-oncologic patients in an independent cohort. In our initial cohort, the sensitivity, specificity, positive predictive value (PPV), and negative predictive value (NPV) associated with EV miR-21 level of <0.25 copy/EV were 85%, 100%, 100%, and 93%, respectively (Figure **6A**). We calculated a need for CSF from 30 additional patients (with 15 glioblastoma patients and 15 non-oncologic patients) for statistical testing of our hypothesis, with type I error set at 0.05 and a power of 0.8 [65]. 

Through a collaborative network with Emory University and the University of California San Diego Neurology programs, 29 additional CSF samples were secured (with 15 glioblastoma specimens and 14 non-oncologic specimens). EVs were isolated from these specimens, and miR-21 levels were determined. In this second and independent cohort, setting EV miR-21 level of <0.25 copy/EV as the discriminating threshold for glioblastoma versus non-oncologic patients yielded sensitivity, specificity, PPV, and NPV of 87%, 93%, 87%, and 86%, respectively (Figure **6B**). Receiver Operating Characteristics (ROC) curves for CSF EV miR-21 as a biomarker of glioblastoma tumor presence show an AUC of 0.9 and p<0.01 (Figure **6C**). In aggregate, these results support CSF EV miR-21 as a feasible biomarker for the presence of glioblastoma. 

In aggregate, these results suggest that CSF EV miR-21 constitutes a biomarker for the presence of glioblastoma cells. A corollary of this prediction is that surgical excision of glioblastoma should be associated with a decrease in CSF EV miR-21 level. We tested this prediction in a patient who underwent CSF sampling at the time of glioblastoma surgery and then at three months after a gross-total resection. Consistent with our hypothesis, the relative abundance of miR-21 in CSF EV was decreased by approximately 50-fold after surgical resection (Figure **6D**).

## Discussion

Analysis of genetic material within glioblastoma secreted within EVs in bio-fluids represents a unique opportunity for diagnosis and therapeutic monitoring. EVs isolated from bio-fluids can encompass exosomes, microvesicles, retrovirus-like particles (RLPs) and apoptotic bodies [66,67]. While the mechanisms of their biogenesis may differ, genetic materials from their originating cells have been detected in all types of vesicles. Enrichment of miRNA in EV content [9] renders EVs a particularly attractive platform. 

The quantitative assessment of EV miRNAs requires thoughtful considerations. Our study reveals that commonly used reference transcripts (GADPH mRNA, 18S rRNA, and hsa-miR-103) were present at extremely low and variable levels in EVs. Moreover, there were no consistent correlations between the level of these transcripts and EV RNA yield or EV particle numbers (Figure **3** and Figure **2**). When comparing the EV particle numbers and the total amount of RNA extracted, we noted a strong correlation in systems such where there is only one source of EV secreting cell (e.g. neurosphere culture; Figure **3A**). Under these conditions, RNA yield serves as an effective normalization method in place of EV number (Figure **7A and 7B**). However, in more complex samples such as patient CSF, where EVs are likely secreted by multiple cells of origin, the correlation between EV number and total RNA yield are significantly lowered (Figure **3C**). Because of these observations, we recommend quantifying EV miRNA in absolute terms and normalizing to total EV number. 

Using this method, we showed that EV miR-21 levels can be used to differentiate CSF isolated from glioblastoma patients and CSF from non-oncologic patients. Importantly, our result was validated using multiple independent CSF collections. It is important to note that CSF is not routinely collected during the course of treatment for glioblastoma patients in the U.S. Thus, while the total number of CSF samples analyzed here is relatively small, with a total of 28 glioblastoma CSF and 28 non-oncologic CSF samples analyzed, our study represents an exhaustive analysis of CSF specimens collected by four independent investigators over a multi-year period. The results presented here provide a sound basis for a prospective, multi-center study to validate CSF EV miR-21 as a biomarker for assessing the presence of glioblastoma.

Recent studies suggest that circulating miRNA can exist in two compartments: 1) outside of EV where they complex with Argonaut2, the catalytic component of the RNA-induced silencing complex (RISC) or high density lipoproteins; and 2) within EVs [68,69]. We add the finding that the majority of CSF miR-21 is of EV origin. Additionally, the available data suggests that genetic material within EVs are quite stable in the CSF [70]. Thus, isolating CSF EV may enhance the sensitivity of CSF miRNA based biomarker assays. 

miR-21 was previously reported as a CSF biomarker for glioblastoma burden in a prior study [70], While our overall conclusion is generally consistent with this previous study, it is worthwhile noting the differences between the two. First, Teplyuk et. al. [70] measured the level of miR-21 in total CSF and not CSF EVs. Second, in the previous study, quantitative assessment of miRNA in the CSF was performed using the relative C_T_ methods using miR-24 and miR-125 as reference transcripts. We tested whether miR-24 and miR-125 are adequate reference transcripts for quantitative EV miRNA analysis. While we observed modest to strong correlations between miR-24/miR-125 expression and EV RNA yield and/or total EV number in cells cultured under neurosphere conditions (Figure **8A**), such correlations were not found in clinical CSF specimens (Figure **8B**). Using criteria established in our study, these results would suggest that miR-24 and miR-25 are not adequate reference transcripts for the quantitative miRNA analysis of CSF EVs. Consistent with this proposition, Teplyuk et. al. reported that miR-24 and miR-25 were present in relatively low levels and varied between clinical specimens [70]. 

miR-21 expression is not unique to glioblastoma, it has been detected in endothelial cells [71], normal breast tissue [72], cervical tissue [73], and hematopoietic cells [74]. While the expression level of miR-21 in glioblastoma cells are generally one to two orders of magnitude higher than those found in normal tissue [34], it is certainly conceivable that the normal cellular secretion of EV miR-21 may mask the presence of a small number of glioblastoma EVs containing miR-21. When EVs isolated from sera were examined, this appeared to have been the case, as no significant differences in EV miR-21 levels in could be detected between patients with and without glioblastoma (Figure **5A**). However, Wang et. al. previously reported that plasma levels of miR-21 were significantly altered in glioblastoma patients compared to normal controls [75]. In the context of our study, it is possible that EV independent miR-21 significantly differed between glioblastoma and non-oncology patients. It is also worthwhile noting the technical difference in the method of miRNA quantitation between our study and this previous study [75]. In the previous study, Wang et al. [75] quantitated miR-21 using the relative C_T_ method, with murine miR-29 as spike-in control. In contrast, our study quantitated miR-21 in absolute terms and normalized to the number of EVs. Both methods harbor inherent advantages and disadvantages [76]. The optimal method of miRNA quantitation in bio-fluids remains an unresolved issue and may be bio-fluid dependent. 

In contrast to our sera derived results, miR-21 levels in the CSF of non-oncologic patients were extremely low by comparison. The amount of miR-21per EV derived from the CSF of glioblastoma patients was, on average, ten-fold higher than those derived from non-oncologic patients (Figure **5B**). These results suggest that the normal tissues in contact with CSF secrete less miR-21/EV as compared to brain tissue harboring glioblastoma cells.

Our studies raised several important questions. Although EVs in CSF appear to be enriched for miR-21, we have little data on the sub-speciation of these EVs in terms of size, zeta potential, shape, or type [67]. Equally uncertain is the origin of CSF EV miR-21, which may be from glioblastoma or associated endothelial, ependymal or inflammatory cells [77,78]. To the extent that we consistently observed the secretion of miR-21 containing EVs from all glioblastoma cells examined, we propose that these vesicles contribute to the presence of hsa-miR-21 in the CSF. However, this remains a point of uncertainty. Finally, with the emergence of the concept of glioblastoma subtypes [79], whether subtyping can be achieved through CSF EV miRNA analysis remains an important question, 

In sum, our study delineates miR-21 levels in absolute terms as number of copies per EV and provides data suggesting the utility of CSF EV miRNA analysis as a biomarker for glioblastoma patients. It is likely that other glioblastoma EV miRNAs [80] may be similarly exploited as diagnostic biomarkers. 

## Supporting Information

Figure S1 **Electron micrograph of EVs isolated from CSF.** EVs were isolated from CSF by differential centrifugation and analyzed by transmission electron microscopy. Scale bar represents 200nm in (A) and 100nm in (B). EVs in the size range of 50-250nm were observed.
(EPS)

Figure S2 **Relative abundance of GAPDH, 18S RNA, and miR-103 in EV derived from sera of glioblastoma and non-oncologic patients.** GAPDH, 18S rRNA, and hsa-mir-103 transcript copy number was plotted against the total number of EVs isolated for RNA extraction. The relative abundance of these transcripts in EV ranged between 1 transcript in 7 EVs to 1 transcript in 106 EVs.
(EPS)

Figure S3 **Correlation between EV number and RNA yield.** For each sample, the RNA yield from EV preparations for (A) neurosphere cell lines, (B) Serum samples, and (C) CSF samples were plotted on the X-axis; the input EV number for these two datasets were plotted on the Y axis. There was excellent correlation between the RNA yield and the EV preparation in neurosphere lines, the correlation was poor in serum samples.
(EPS)

Figure S4 **Abundance of EVs per cc of biofluids.** (**A**) Abundance of EVs per cc of serum in glioblastoma and non-oncogenic patients. There was no significant difference between tumor and control samples. (**B**) Abundance of EVs per cc of CSF in glioblastoma and non-oncogenic patients. No significant differences were observed.
(EPS)

Figure S5 **Average size profile of EVs isolated from biofluids.** (**A**) Average size distribution of EVs isolated from serum of glioblastoma and non-oncogenic patients. There was no significant difference between tumor and control samples. (**B**) Average size distribution of EVs isolated from the CSF of glioblastoma and non-oncogenic patients. No significant size differences were observed.
(EPS)

Figure S6 **Effects of culturing condition on miR-21 expression in cells and EVs.** Glioblastoma cell lines U87MG and CMK3 were grown under adherent or neurosphere condition and the miR-21 level in cells or EVs were quantitated.
(EPS)

Figure S7 **Normalization of EV miR-21 by RNA yield.** (**A**) The expression level of miR-21 was quantitatively assessed in nine neurosphere glioblastoma lines using total RNA yield as normalization parameter. (**B**) Comparison of normalization method revealed good correlation between normalizing by EV particle number or total RNA yield in cell culure. (**C**) Levels of miR-21 in EVs isolated from glioblastoma and non-oncologic patients normalized by RNA yield. (**D**) Correlation between normalization by EV number or total RNA yield in CSF.
(EPS)

Figure S8 **Relative abundance of miR-24 and miR-125 in EVs derived from neurosphere glioblastoma cell lines and CSF of glioblastoma and non-oncologic patients.** has-miR-24 and has-miR-125 levels in EV isolated from from (**A**) 9 glioblastoma neurophere lines, cultured under serum-free conditions or (**B**) from CSF of 13 glioblastoma and 14 non-oncologic patients were plotted against total RNA extracted from the EVs or against the total number of EVs isolated for RNA extraction.
(EPS)

Table S1 **List of primers used.**
(DOCX)
