# Supplementary material for: miR-21 in the Extracellular Vesicles (EVs) of Cerebrospinal Fluid (CSF): A Platform for Glioblastoma Biomarker Development
Source: PLoS One. 2013 Oct 21;8(10):e78115. doi: 10.1371/journal.pone.0078115 (PMC3804457; doi:10.1371/journal.pone.0078115)
Supplement: Table S1 — List of primers used. (DOCX) [file pone.0078115.s009.docx]

**Supplemental Table 1: List of primers used**

|  | Forward Primer | Reverse Primer |
| --- | --- | --- |
| GAPDH | ACCCAGAAGACTGTGGATGG | TTCTAGACGGCAGGTCAGGT |
| 18S rRNA | GGGAGGTAGTGACGAAAAATAACAAT | TTGCCCTCCAATGGATCCT |
| miR-21 | LNA^TM^ PCR primer set, Product No. 204230 | |
| miR-103 | LNA^TM^ PCR primer set, Product No. 204063 | |
| miR-24 | LNA^TM^ PCR primer set, Product No. 204260 | |
| miR-125 | LNA^TM^ PCR primer set, Product No. 204465 | |
